# Why is it so hard to enact responsible change? Scientists need to work more closely with other social groups to implement sustainable innovation

**DOI:** 10.15252/embr.201949493

**Published:** 2020-03-09

**Authors:** Ceilidh Welsh, Lydia Pike, Jonathan Elliott, Jonathan Bailey, Rachael Quintin‐Baxendale, Jessica Billington, Adriano Matousek, Chloe Matthews, Dragos Dumitrescu, John Felipe Murphy, Mark Hewlett, Chloe Singleton, Paul James, Sarah Hartley, John Love

**Affiliations:** ^1^ University of Exeter Exeter UK

**Keywords:** S&S: Ethics, S&S: Economics & Business

## Abstract

Science is key to developing sustainable products and solutions. But scientists also need to work more with governments, industry and society to help implement those solutions.

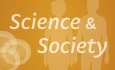

It is tempting to think that once scientists develop a solution to an environmental problem that ticks all the right boxes, it will automatically change the world for the better. In reality, however, this is rarely the case. Good ideas often remain completely ignored by society, while the machine of industry rumbles on, unfettered by well‐meaning attempts to steer it towards a more ecologically friendly track. In fact, there are many more factors that determine the success of a good idea to solve environmental problems than just scientific merit. What is it then that prevents society from enacting widespread environmental change despite the many good ideas emanating from research? And what can scientists do about it?

… there are many more factors that determine the success of a good idea to solve environmental problems than just scientific merit.

There is no short answer to these questions, but ultimately it boils down to the intrinsic value we place on the environment and the complexities of our economic, political and social systems. Time and again, we encounter obstacles to enacting environmental change, often because industry places a higher value on short‐term profits than on long‐term sustainability, or owing to a lack of regulation by governments, or resistance by consumers to adopt a new product. Possibly the most obvious example is the case of climate change. For years, energy corporations have hindered global and national efforts to reduce greenhouse gas emissions; governments have not introduced enough effective regulations that would help to curb the continued release of greenhouse gases; and the public has struggled to change their consumption habits.

## Shifting responsibility

However, as scientists, we cannot just blame other societal groups but need to consider what we can do to facilitate change. Instead of acting independently from industry, government and society, we need to recognise that we must work together to achieve sustainable development. As a team of undergraduate scientists, we encountered similar roadblocks when developing a solution for tackling microplastic pollution within our environment. We designed an enzyme‐based filter system for laundry machines that captures microplastic fibres that are released from synthetic fabrics—35% of all microplastics released into our environment come from synthetic clothing 1.

While large companies in both fashion and appliance industries showed interest in our invention, the feedback we received shifted the responsibility for tackling microplastic pollution down the chain to other industry sectors, and ultimately the consumer. This was so subtle that we did not initially recognise it. Companies could not immediately see the long‐term benefits of integrating microplastic capturing systems into their laundry machines, citing efficiency or economic drawbacks. We therefore aimed to design an external filtration system that consumers could buy and add to their laundry machine. However, this solution shifted the responsibility for protecting the environment from the clothing industry and laundry machine manufacturers to the consumer, who must pay the additional price.

A solution that places responsibility on the consumer does not truly reflect the realities of environmental pollution. We all contribute to climate change and environmental damage through our actions, but some contribute more. One hundred companies have been the source of more than 70% of the world's greenhouse gas emissions since the 1980s 2, but these companies have also supplied most of the energy to sustain our modern lifestyles.

A solution that places responsibility on the consumer does not truly reflect the realities of environmental pollution.

Through our interdisciplinary engagement, it became apparent that science, industry and government need to work together with the general public and stakeholders to find innovative and cost‐efficient solutions to environmental problems. As industry is ultimately driven by profits, we must consider ways to maintain their profitability without putting the onus on the consumer. These interactions can be facilitated most effectively using a responsible innovation framework developed by scientists who specialise in the interaction between scientific innovation and its integration in society. Responsible science must consider “the limitations of current approaches to governance, [and call] for the full range of actors, including diverse public stakeholders, to carefully consider the economic, political and social context of environmental problems and solutions as well as the social and ethical concerns” 3.

## Cooperation is key

Indeed, some of the best examples of reducing plastic pollution highlight how legislation, business and consumers can efficiently cooperate. In October 2015, the UK government introduced a five pence plastic bag surcharge 4 that was quickly implemented by supermarket chains; the sales of plastic bags in the UK's top seven supermarket chains decreased by approximately 90% 5, as people switched to reusable bags for shopping. This is a prime example of cohesive and productive collaboration between all sectors: government enacts regulation, which creates a level playing field for business and leads to a change in consumer behaviour. So how can scientists fit into this collaborative effort?

Scientists can cooperate with industry to develop environmentally friendly products or systems that are economically attractive for retailers and manufacturers.

As scientists, we need to become more aware of the economic, political and social factors that shape change. With greater knowledge of these factors, we can better understand the problems we are trying to solve and adapt our approaches to translate research into innovation with government, industry and the general public. It is important to recognise that environmental responsibility does not lie with one societal group, even if some solutions are inevitably engineered to target one such group. Clear two‐way communication, with honest facts and hard‐to‐stomach truths, should be prioritised so that collaborative efforts can target the real underlying problem, not superficial symptoms. Scientists can ultimately play a crucial role with and for each of these societal groups to encourage the adoption of environmental solutions. It is important to recognise where our involvement is most beneficial and efficient, to ensure the impact of the environmental solutions we develop.

Within government, scientists can provide solid evidence for drafting legislation and predicting its impact and efficiency. For example, Tamara Galloway (University of Exeter, UK), who studies the effects of microplastics on marine life, collaborated with SAPEA (Science Advice for Policy by European Academics, an organisation that provides scientific advice to the European Commission) to discuss legislation surrounding cosmetic microbeads in the UK 6. Galloway and other researchers provided evidence to a cross‐party committee on the environment, which eventually led to a ban on cosmetic microbeads, preventing thousands of tons of these microplastics reaching the oceans every year.

Scientists can cooperate with industry to develop environmentally friendly products or systems that are economically attractive for retailers and manufacturers. For example, the development of the blue LED—rewarded with the 2014 Nobel Prize in Physics for Isamu Akasaki, Hiroshi Amano and Shuji Nakamura—finally enabled the white LED as an alternative to filament and incandescent light bulbs. As LEDs require five times less energy than conventional light bulbs and last much longer, they have enormous potential for saving energy and reducing the amount of CO_2_ released in the atmosphere. This prompted the EU ban of non‐directional halogens in 2018 7. Since then LEDs have become highly popular among consumers given their energy efficiency and longer lifespan. Moreover, LEDs enabled industry to develop a wide range of novel and innovative products.

It shows that if scientists cooperate with industry to enable companies to make profits in the long term and improve their brand image, there is a greater chance that new, environmentally friendly products will find their way to the market. Equally, industry and business are always looking for a competitive edge, using their Research and Development (R&D) departments to develop new products that align with consumer wants and needs. If scientists collaborate with industry's R&D departments, it increases the chance of new innovation that will not only benefit business but also benefit the environment too. Akasaki, Amano and Nakamura dedicated so much of their time and efforts to developing the blue LED because they knew it was the missing piece for the white LED and therefore key to developing energy‐efficient lighting.

## Involving society

Scientists can cooperate with different societal groups too. The recent interest in co‐production of research has highlighted numerous examples of interaction with stakeholders leading to effective solutions to environmental problems. Working with stakeholders helps scientists understand the complexity of the problem they are trying to solve and how solutions might become more effective. Co‐producing knowledge increases the likelihood that such knowledge will be used in decision‐making, whether by citizens, stakeholders, communities or policy‐makers.

Working with stakeholders helps scientists understand the complexity of the problem they are trying to solve and how solutions might become more effective.

These scientific activities should not occur in isolation as the main societal groups cannot function, or even exist, without the others, even if each group successfully contributes within our society. Governments should encourage industry to implement and integrate greener solutions through legislative action, tax breaks, subsidies or fines. Businesses should be able to make a profit to remain competitive and innovative. Sharing research results within industry R&D departments and academic institutions could further encourage the development of responsible advancements with many alternative viewpoints and give industry the scientific expertise and endorsement to develop sustainable services and products.

Scientists need a greater understanding of the systemic problems that prevent their academic research from turning into practical solutions …

Moreover, society should be more involved. People care about climate change and the planet's future, so their perspective should be considered when developing new products and services that affect their daily lives. Scientists should continually engage with each group, providing evidence, sharing their research and listening, to bring awareness to environmental issues. The interconnection between societal groups and scientists brings a variety of knowledge and different levels of authority to a large‐scale problem, which can help to increase the likelihood of developing successful solutions to our contemporary problems.

Maybe in today's world, the view of a scientist as someone wearing a white lab coat in a university laboratory is no longer enough. Scientists need a greater understanding of the systemic problems that prevent their academic research from turning into practical solutions and need to choose the best form of intervention to enable these solutions to be realised. As this world is our shared heritage, all societal groups should share the responsibility of enacting changes that will benefit everyone in the long term.
